# Language can mediate eye movement control within 100 milliseconds, regardless of whether there is anything to move the eyes to

**DOI:** 10.1016/j.actpsy.2010.09.009

**Published:** 2011-06

**Authors:** Gerry T.M. Altmann

**Affiliations:** Department of Psychology, University of York, UK

**Keywords:** Oculomotor control, Saccades, Double-step paradigm, Language-mediated eye movements, Visual world paradigm

## Abstract

The delay between the signal to move the eyes, and the execution of the corresponding eye movement, is variable, and skewed; with an early peak followed by a considerable tail. This skewed distribution renders the answer to the question “What is the delay between language input and saccade execution?” problematic; for a given task, there is no single number, only a *distribution* of numbers. Here, two previously published studies are reanalysed, whose designs enable us to answer, instead, the question: How long does it take, as the language unfolds, for the oculomotor system to demonstrate sensitivity to the distinction between “signal” (eye movements due to the unfolding language) and “noise” (eye movements due to extraneous factors)? In two studies, participants heard either ‘the man…’ or ‘the girl…’, and the distribution of launch times towards the concurrently, or previously, depicted man in response to these two inputs was calculated. In both cases, the earliest discrimination between signal and noise occurred at around 100 ms. This rapid interplay between language and oculomotor control is most likely due to cancellation of about-to-be executed saccades towards objects (or their episodic trace) that mismatch the earliest phonological moments of the unfolding word.

## 1 Introduction

Until recently, interest in the speed with which saccades can be launched towards intended (or unintended) targets has been restricted to the domain of visual psychophysics. Studies of oculomotor capture (e.g. Theeuwes, Kramer, Hahn, & Irwin, 1998), as well as of the distinction between anti- and pro-saccades (Hallett, 1978), have informed estimates of the time it takes to plan and then launch a saccadic eye movement. Studies of how launch times are influenced by the *likelihood* that a target to which the eye should saccade will appear in one position or another, have also informed theories of the time-course with which information apprehended from the visual environment can drive the oculomotor system to launch a saccade (Carpenter & Williams, 1995). Unsurprisingly, there has been some considerable convergence on the properties of this time-course. Most estimates assume that the time to plan and then launch an eye movement varies between 150 and 200 ms. Interestingly, these estimates have not changed significantly since the earliest estimates from the 1930s (Travis, 1936). The present paper also explores the speed with which language input can modulate oculomotor control, but in the context of the ‘visual world’ paradigm (Cooper, 1974; Tanenhaus, Spivey-Knowlton, Eberhard, & Sedivy, 1995), in which participants' eye movements are monitored as they listen to instructions to manipulate objects presented within their visual environment (either real objects or objects depicted on a computer screen), or as they listen to sentences or narratives that describe events which may affect the people and/or objects depicted in a concurrent (or previously seen) scene. The paradigm presents a number of challenges to estimates of saccadic launch times: For example, participants are not waiting at a pre-determined location for a GO signal as the dynamically changing, and noisy, linguistic signal unfolds – rather, they have shifted their gaze to a new location (for a variety of reasons not all of which are determined by the linguistic signal) and are engaged in one or more of apprehending information, shifting their attention, planning their next saccade, or even executing that saccade, as the linguistic signal unfolds. The interplay between the unfolding language and the saccadic control system(s) is therefore particularly noisy (most regulatory neurophysiological systems are inherently noisy, but psychophysical studies of oculomotor control minimize the noise), and a distinction therefore has to be drawn between eye movements due to the language signal itself, and those due to extraneous noise; this allows the critical time-course issue to be recast in terms of the time-course with which the saccadic system exhibits a distinction between signal-driven and noise-driven saccades, where the signal to move the eyes to a visual target is an unfolding word that refers to that target. It is this issue that will be the focus of the present article.

The speed with which the language signal can influence the oculomotor system is relevant within the visual world paradigm because there is necessarily a delay between the cognitive system entering some state or other, and the *consequence* of that change in state manifesting in the eye movement record; the unfolding language may induce that change in cognitive state but when can we expect to see that change reflected in the eye movements? More commonly, the visual world paradigm requires an answer to the reverse question: Given a shift in eye gaze, when did the cognitive system enter the state that *caused* that shift in gaze? And which point in the unfolding language triggered this change in state?

For the purposes of exposition it does not matter whether the changes in state to which we refer are gradual or catastrophic, or whether there is one precise point in the language that effects this change, or whether there is a gradual accrual of information from the language that modulates cognitive states across time. For expository purposes, these changes will be described as if they are punctual, although most likely they are not, given contemporary models of the interplay between information accrual and the decision to launch an eye movement (Carpenter & Williams, 1995; Reddi, Asrress, & Carpenter, 2003; Reddi & Carpenter, 2000) and models of cognition in which knowledge is distributed within a dynamical system across a representational substrate supporting spreading activation (cf. Elman et al., 1996; Rogers & McClelland, 2004; Rumelhart & McClelland, 1986). Indeed, it is precisely because the events of interest (whether in the language or in the cognitive system) are not punctual that the work described below adopts a *distributional* analysis of the data, which assumes an underlying stochastic mapping between language and cognitive control of the oculomotor system (and which assumes that the mapping between language and cognitive control more generally is itself stochastic).

A study by Allopenna, Magnuson, and Tanenhaus (1998) exemplifies the issues regarding the time-course with which language can mediate eye movement behaviour in the visual world paradigm. Allopenna et al. monitored eye movements as participants viewed a display depicting a beaker and a beetle. Their interest was in establishing the profile of eye movements towards these two objects as the instruction ‘pick up the beaker’ unfolded. More specifically, they were interested in the time-course with which the eye movement record would distinguish between looks to the beaker and looks to the beetle (as well as looks to a speaker). To begin with, eye movements (across participants and trials) were directed towards both objects, because the unfolding speech wave was compatible with both the words ‘beaker’ and ‘beetle’. At some later point, the eye movements would reflect the distinction between these two alternative possibilities, with increased fixations on the beaker, and reduced numbers of fixations on the beetle. Allopenna et al. (1998) reported fixation probabilities across time in 33-ms intervals, scoring each interval according to which object was being fixated during that interval. Fixations on the beaker began to distinguish from fixations on the beetle at around 400 ms post onset. Even earlier, the beaker and beetle were fixated more often than the distractor object (a baby carriage) beginning around 200 ms from word onset.

Although Allopenna et al. reported their data in fixational terms, they in fact calculated the onset of a fixation not in terms of when the eyes first landed on the target, but in terms of when the eyes initiated the saccade which *culminated* in landing on that target (Magnuson, personal communication). This is particularly relevant when estimating the time-course of the cognitive events that modulate eye movement control. Had they calculated fixation onsets in terms of when the eyes landed (which some investigators do), they would have over-estimated this time-course: Saccadic durations depend on the distance the eye travels from one fixation location to the next (and in practice, when analysis is automated, they depend also on the precise algorithms used to parse the sampled eye gaze position into saccades and fixations); it is not atypical to observe distributions ranging from 0 to in excess of 100 ms, and thus the onset of the saccade that lands on the target is a better estimate, compared against actual landing time, of when the cognitive system entered the state that caused the movement towards the target. It is a better estimate not simply because it is closer in time to that event, but because the noise introduced through variable saccadic durations is eliminated. Allopenna et al.'s findings suggest that it took around 200 ms from when the acoustic signal could *in principle* first modulate the eye movement patterns towards just those objects in the scene whose names matched the earliest moments of the unfolding signal, to when it *actually* modulated that pattern: Information at and immediately after word onset resulted, 200 ms later, in a greater preponderance of eye movements towards just those objects in the display whose names started with the same initial sounds as the unfolding word.

The delay just described consists of at least two conceivably dissociable components: The interpretation of the acoustic signal in the context of the scene, and the time it took to plan and subsequently *initiate* the eye movement. In fact, whether these components are indeed dissociable is itself debateable – interpretation and planning are not so easily dissociated in accounts of interpretation in which (i) interpretation proceeds through the modulatory influence of sensory information on the internal state of the system (as is common in connectionist and other dynamical systems), (ii) the modulatory influence of one representation (e.g. linguistic) on the selection/processing of another (e.g. visual) constitutes an attentional process in which some representations are favored (i.e. differentially activated) over others (cf. the *biased competition theory* of Desimone & Duncan, 1995, and *the guided activation theory of cognitive control* of Miller & Cohen, 2001), and (iii) shifts in covert visual attention (i.e. differential activation of one object representation relative to another) constitute the saccadic plan (cf. *the premotor account of attention*; e.g. Rizzolatti, Riggio, Dascola, & Umiltá, 1987). Within the framework described by these different accounts of cognitive control, attention, and motor planning, the relationship between interpretation and planning is akin to an incremental or cascaded process without a definitive boundary separating one from the other – planning *is* interpretation, and vice versa. The picture is further complicated by the fact that programming of more than one saccade (and all that that entails) can occur in parallel (e.g. Becker & Jurgens, 1979; Walker & McSorley, 2006) Whether separate interpretive and planning stages are assumed, or whether they are instead inextricably inter-twined and linked, it is still possible to ask how long it can take between an external signal and the initiation of a saccadic eye movement (the *saccadic latency*). As will become apparent, the estimate based on Allopenna et al. (1998), that it takes 200 ms to apprehend the signal, plan a saccade consequent on the content of that signal, and execute that saccade, does not necessarily translate into a 200 ms delay between signal apprehension/interpretation and modulation of oculomotor control. Indeed, the data presented below suggest a lower bound on this modulation of approximately 100 ms.

## 2 Estimating saccadic latencies

Early studies on the time-course of signal apprehension and saccadic planning involved eye movement studies in which participants fixated successive locations indicated by a range of visual stimuli, including lighted bulbs (Saslow, 1967) and fixation crosses (Rayner, Slowiaczek, Clifton, & Bertera, 1983). These studies measured the delay between stimulus onset and the initiation of the saccade towards the target, with latencies in the 200–220 ms range. Saslow (1967) observed a reduction in the launch latency if the participant knew in advance the location of the stimulus to which they would have to move their eye (see Carpenter & Williams, 1995, for an account of how such a reduction may come about). In a later study, Matin, Shao, and Boff (1993) attempted to determine the ‘saccadic overhead’ – in effect, the time it takes to plan and launch a saccade minus the time taken to process/apprehend the visual stimulus. That study in fact estimated an overhead of around 100 ms, based on a procedure in which they subtracted between conditions which either did or did not require a saccadic eye movement in order to view and judge the visual stimuli they presented to their participants. However, they did not monitor eye movements directly, but rather based their estimates on participants' response times in a push-button judgment task. The current consensus on the time it takes to process a triggering cue (i.e. the cue to move the eyes), and programme a new eye movement indicates programming times of 150 to 200 ms (e.g. Becker, 1991; Findlay, 1997; Salthouse & Ellis, 1980). Programming and execution times in studies of oculomotor capture of eye movements (e.g. Theeuwes et al., 1998) are typically just over 200 ms.

In the visual world paradigm, unlike in the majority of studies that have estimated saccadic latencies, the trigger to move the eyes towards a target is not the visual target itself, but the unfolding language, with the precise content of the language determining the identity of the object to which the eyes should be oriented. In the one study that attempted to estimate saccadic latencies in the visual world paradigm (Altmann & Kamide, 2004), a range of different estimates were arrived at, depending on whether the cue to move the eyes was a word which also cued the identity of the target object to which participants had to move their eyes, or whether the target identity was known in advance. Median launch times to the named target (see below) were just under 500 and just over 200 ms respectively. However, even in these cases, the eyes were in a pre-determined location prior to the critical saccade – that is, in this study (like the others based on visual triggers), there was minimal noise due to the oculomotor system being “in flight” (i.e. already shifting attention and gaze due to a combination of exogenous and endogenous factors by the time of the trigger; see below).

Notwithstanding the fact that estimates of saccadic latency have not, to date, taken account of the noisy conditions that prevail in the visual world paradigm, a significant number of visual world studies have assumed an average saccadic latency of 200 ms as a lower bound on when information from the unfolding speech stream first modulate the eye movement system. Many of these studies have then used this estimate to “resynchronize” the eye movement record with the speech stream. In the context of the Allopenna et al. (1998) study (they did *not* resynchronize in the manner described here, but their study is used here purely for the sake of exposition), this would involve analyzing eye movements not from word onset, but rather from 200 ms *after* word onset. The logic is that because it (apparently) takes a minimum of 200 ms between signal and eye movement, any eye movement launched between word onset and this 200 ms point could not have been driven by the acoustic input within that 200 ms period. So eye movements observed at, say, 200 ms after the onset of the signal would be reinterpreted as being due to “events” at signal onset. Applying this logic to the Allopenna et al. findings yields a surprising result: By 200 ms post onset, the eyes were looking more towards the beaker and speaker than towards the other objects in the display – the saccades that yielded these data 200 ms post onset were initiated by cognitive events that occurred, therefore, *no later* than word onset!

This last observation suggests that we should revise downwards our estimate of saccadic latencies, and attempt to establish what actually *is* the earliest time in which we could see language-mediated influences on the eye movement record. In Altmann and Kamide (2004), we pointed out that the distribution of launch latencies in the visual world paradigm can, depending on the conditions, be highly skewed (and this mirrors the general finding that behavioral responses of any kind tend to be skewed). For example, when the cue to move the eyes to a pre-determined object was a tone, the mean launch latency was 254 ms, the median latency 181 ms, and the modal latency 132 ms. This suggests that there can be no single numerical answer to the question “how long does it take for the unfolding language to influence the eye movement record?” – the answer is a *distribution* (and hence the inappropriateness of “resynchronizing” the eye movement record on the basis of the logic described above – the skewed distribution underlying the mean tendency means that a very significant number of eye movements will certainly have been influenced by the linguistic signal in less time than the mean saccadic latencies derived from the earlier psychophysical studies). In Altmann and Kamide (2004), we asked: How long does it take between the onset of a signal to move the eyes and the onset of the corresponding movement? – This was, after all, the same question that had been asked in many of the preceding studies of saccadic launch latency. A different question (which we did not ask in Altmann & Kamide, 2004) is perhaps more appropriate: When does the eye movement record first indicate discrimination, on the basis of the linguistic input, between the alternative objects to which the eyes can be targeted? Although it might seem that Allopenna et al. have already answered this question (200 ms), in fact, they have not (this is not a criticism of the Allopenna et al. study – it was not designed to address this particular question): Some of the time, in the Allopenna et al. study, the eyes will have launched towards the beaker or the speaker because those launches were indeed *signal driven*. But some of the time, they will have launched towards those two objects for other *extraneous* reasons that were independent of the content of the linguistic signal. Similarly, some of the times that they launched towards the distractor object (the baby carriage) may not have been due to the signal being ambiguous still, but may have been due to whatever extraneous factors cause the eyes to move to one object when the acoustic signal unambiguously refers to another. This means that determining the precise point at which the system *first discriminates* between the alternative objects on the basis of the acoustic signal is complicated by a confounding of *signal-driven* eye movements with what can termed *noise-driven* eye movements. This noise can mask the point at which signal-driven eye movements do in fact discriminate between the alternative objects in the display. Some of the noise can be due to comparing looks to one object with looks to another (in which case extraneous differences between the objects, in color, texture, size, shape, or identity, might make one more attractive than another); or due to comparing looks in response to one spoken stimulus with looks in response to another (differences in the unfolding acoustic–phonetics can influence the degree to which one signal can more effectively cue its referent than another).

In the remainder of this paper, a method is described for distinguishing between *signal* and *noise* in the eye movement record that avoids these complicating factors. Data are then reported from two separate studies which converge on the same estimate of when language can effect, via the oculomotor system, discrimination between alternative targets. Participants in these two studies were not constrained to look at a fixation point (and indeed, their eyes could be fixating anywhere within the scene prior to the onset of the target word) and they were given no overt task to perform other than to “look and listen” (this task has been discussed extensively elsewhere). In one of the two studies, the visual scene was removed (and the screen remained blank) before the onset of the target word. But the result was the same: Language-mediation of oculomotor control occurred in both studies within 100 ms of the onset of the word that determined the appropriate target to fixate.

## 3 Distinguishing signal from noise

**Fig. 1:**
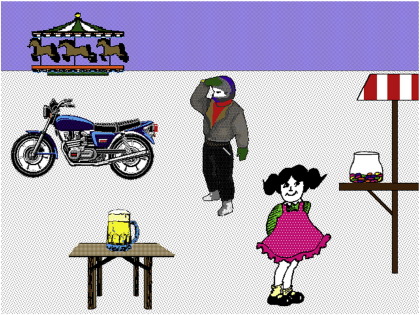
Example stimulus from Kamide et al. (2003).

The data reported below are based on re-analyses of studies reported by Kamide, Altmann, and Haywood (2003) and by Altmann (2004). Kamide et al. showed participants scenes such as the one shown in Fig. 1. One second after the onset of the scene, participants heard one of the following four sentences:

(1) The man will ride the motorbike
(2) The girl will ride the carousel
(3) The man will taste the beer
(4) The girl will taste the sweets

We reported in that study eye movements initiated from the verb onwards (given the questions that we were addressing at that time). In the reanalysis described below, however, eye movements are calculated towards the man or the girl after hearing ‘man’ or ‘girl’. One can ask of these data how long it takes for the eyes to move to the man after hearing ‘man’, or to the girl after hearing ‘girl’. However, instead of calculating an average saccadic latency, the distribution of latencies will be calculated. This distribution can then be contrasted with the distribution of saccadic launch latencies towards the man after hearing ‘girl’ or towards the girl after hearing ‘man’. The logic is as follows: The first saccade launched after the onset of ‘man’ towards the man might have been a signal-driven saccade (driven by the *content* of the unfolding signal) but it might alternatively have been launched towards the man for reasons that are independent of the signal. The latter would be a “noise-driven” saccade. Saccades launched after the onset of ‘girl’ towards the man will include only noise-driven saccades, and this distribution allows us to estimate the noise component of saccades launched to the man after the onset of ‘man’.^1^ Given these two distributions (signal + noise, and noise only), it is possible to determine not simply the averages of each distribution, but more importantly, the *earliest* point at which the two distributions deviate – the point at which the signal first influences the eye movement record. For example, if we were to count the number of saccades launched within the first 40 ms after noun onset, we would most likely find as many saccades launched to the man after hearing ‘man’ as after hearing ‘girl’, but later on, there will come a point at which more saccades are launched to the man after ‘man’ than after ‘girl’. This pattern would reflect the fact that if probed too early, there will not have been time for the system to interpret the unfolding acoustic input, match or integrate that with knowledge derived from the visual scene (knowledge about the objects and their names), and program and initiate a saccade towards the appropriate object (no claim is being made here about whether interpreting the unfolding acoustic signal and integrating with visually derived knowledge is a staged or continuous, parallel/cascaded, process). If probed later on, however, there would have been sufficient time in which to distinguish that what was being heard mapped onto a specific object depicted in the scene, with appropriate modulation of the eye movement system.

**Fig. 2:**
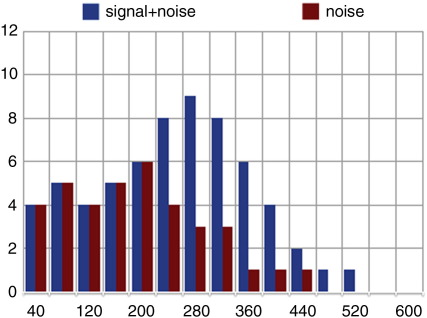
Hypothetical distribution of saccadic launch times. Signal + noise = saccades launched towards the man during the word ‘man’ and towards the girl during ‘girl’. Noise = saccades launched towards the man during the word ‘girl’ and towards the girl during ‘man’. Saccades are plotted in successive 40 ms bins, with the first bin being 0–40 ms. Each bar shows saccades as a percentage of total saccades launched towards the man and girl during the acoustic lifetimes of ‘man’ and ‘girl’ (hence all bars sum to 100).

Fig. 2 exemplifies this logic, and plots two hypothetical distributions of eye movements (signal + noise, and noise) as a histogram depicting absolute numbers of saccades launched within successive 40 ms bins from the onset of either ‘man’ or ‘girl’. To generate these data, all saccades hypothetically launched towards the man were separated into those launched during ‘man’, and those launched during ‘girl’. To these were added, respectively, all saccades launched towards the girl during ‘girl’ and towards the girl during ‘man’. Only the latency to the first saccade launched following word onset is included (on some trials, a second saccade might be launched within the time window defined by the duration of the noun, but these do not change the pattern of latencies in the first few bins showed in the figure; the real data plotted below, in Fig. 4, only change by including subsequent saccades in the tail of the distribution). The data shown in the figure can be interpreted as indicating that in the first five bins (through to 200 ms after word onset), as many saccades were launched towards the man on hearing ‘man’ as on hearing ‘girl’ (and towards the girl on hearing ‘girl’ as on hearing ‘man’ – henceforth, and purely for the sake of exposition, the data will be described as if they simply refer to looks towards the man during ‘man’ or ‘girl’, when in fact they refer also to looks towards the girl during ‘girl’ or ‘man’). In the sixth bin, between 200 ms and 240 ms, the distributions diverge, with more looks towards the man during ‘man’ than during ‘girl’. In effect, this is the first bin at which signal is distinguished from noise. In this example, we would conclude that the cognitive system mediates the eye movement record within 200–240 ms, but that beforehand, the eye movement record was not influenced by the content of the language signal.

An advantage of the designs employed in Kamide et al. (2003) and in Altmann (2004) is that any differences in the launch time distributions cannot be due to low-level visual differences that might make the target object (e.g. the man) to which saccades must be launched more salient than the ‘competitor’ object (e.g. the girl) – for example, if the man were more visually salient than the girl, and this increased salience meant that saccades were launched earlier to the man than to the girl, or more frequently, this increased salience would influence looks to the man both when hearing ‘man’ and when hearing ‘girl’. Similarly, longer launch times or fewer launches to the girl (due to its hypothetically lower salience) would influence looks to the girl both when hearing ‘girl’ and when hearing ‘man’. By combining the two sets of data, any differences in salience are, in effect, subtracted out. The same is true for the manner in which looks to each object are measured in response to both ‘man’ *and* ‘girl’; the same auditory stimulus constitutes, in one case noise, and in the other, signal, and thus low-level acoustic differences are also ruled out as explanations for the data.

In the following sections, the two studies are described from which the data were drawn; relevant procedural details are repeated here from the original studies.

## 4 Study 1

### 4.1 Methods (see Kamide et al., 2003: Experiment 2 for full details)

#### 4.1.1 Participants

Sixty-four participants from the University of York student community took part in this study.

#### 4.1.2 Stimuli

Twenty-four scenes similar to Fig. 1 were each accompanied by the four sentential conditions in (1)–(4). above. There were two versions of each scene depending on the agent of the sentence. Fig. 1 shows the version for when the man was the agent. When the agent was the girl, the positions of these two characters were swapped (their size was adjusted to take account of perspective). The prosody of each spoken utterance was normal, but slightly exaggerated to ensure “clear and careful” speech (there was no gap, however, between the sentence-initial determiner and the subsequent noun). A speech editor was used to mark the onset of the first noun. The mean duration of the first noun was 467 ms. The mean duration of the sentence-initial determiner was 176 ms (see below for discussion of whether coarticulatory information on the determiner, pertaining to the identity of the following segment, might be relevant to the interpretation of the data). The mean duration for the sentence overall was 2075 ms (although only eye movements that were launched during the first noun will be considered).

#### 4.1.3 Procedure

Participants wore an SMI EyeLink head-mounted eye-tracker, sampling at 250 Hz from the right eye (viewing was binocular). The “look and listen” task was employed (see Kamide et al., 2003 for instructions). The onset of the visual stimulus preceded onset of the pre-recorded sound file by 1000 ms.

## 5 Study 2

### 5.1 Method (see Altmann, 2004 for full details)

#### 5.1.1 Participants

Thirty participants from the University of York student community took part in this study.

#### 5.1.2 Stimuli

**Fig. 3:**
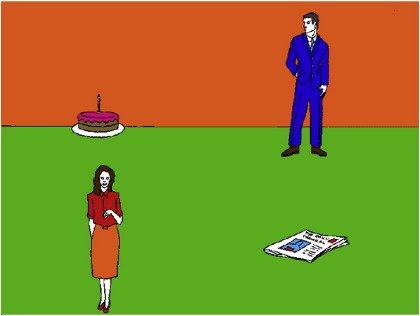
Example stimulus from Altmann (2004).

Twenty scenes similar to Fig. 3 were each accompanied by one of the two sentential conditions:

(5) The man will eat the cake
(6) The woman will read the newspaper

Each scene depicted two protagonists and two items that respectively satisfied the selectional restrictions of the verbs. For half the scenes, the protagonists and items were distributed across the four quadrants as shown in Fig. 3. For the other half, they were in a “diamond” configuration (the upper and lower target items were placed on the vertical midline, and the left and right target items on the horizontal midline). The sentences were recorded using clear and careful speech as in Study 1, and a speech editor was used to mark the onset of the first noun. The mean duration of the first noun was 403 ms. The mean duration of the sentence-initial determiner was 170 ms (again, see below for discussion of coarticulation). The mean duration for the sentence overall was 2543 ms (although again, only eye movements that were launched during the first noun will be considered).

#### 5.1.3 Procedure

The same procedure was employed as for Study 1 except that the scene was presented for 5 s before being removed. One second later, with the screen blank, the auditory sentence was presented to the participant. The “look and listen” task was employed (see Altmann, 2004, for instructions).

## 6 Results

For Study 1, saccades were deemed to have landed on the man or the girl in just those cases that they landed on the actual pixels occupied by the man or girl (no claim is made about the accuracy of eye movements – this criterion was chosen simply to avoid an arbitrary decision about where to place the boundary if not at the object's edge). For Study 2, saccades were calculated by quadrant (or equivalent for the stimuli arranged in a diamond configuration): saccades that landed in the quadrant that had been occupied by the man or the woman were deemed to have been launched “towards” the location previously occupied by these protagonists. This necessarily meant that approximately 25% of the data were lost (because the eye may have already been fixating, through chance alone, within the target quadrant, in which case saccades on that trial were not included in the analyses). Altmann (in press) demonstrates that the pattern and approximate timing of eye movements in this study remains the same regardless of how the regions of interest are defined (quadrants vs. rectangles covering the target objects vs. exact pixels occupied by the objects); however, the absolute number of saccades does change, even if the relative patterns do not, and it is for this reason that quadrant analyses were adopted here (to lessen the sparseness of the data – see below – despite losing 25% of the data in the quadrant analyses, there were still more saccades that could be entered into the analysis than if smaller regions of interest were employed).

**Fig. 4:**
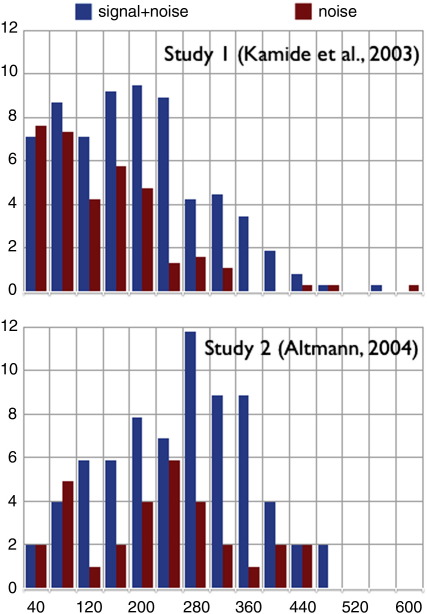
Distribution of saccadic launch times in Studies 1 and 2. Signal + noise = saccades launched towards the man (or its quadrant) during the word ‘man’ and towards the girl during ‘girl’. Noise = saccades launched towards the man during the word ‘girl’ and towards the girl during ‘man’. Saccades are plotted in successive 40 ms bins, with the first bin being 0–40 ms. Each bar shows saccades as a percentage of total saccades launched towards the man and girl during the acoustic lifetimes of ‘man’ and ‘girl’.

Fig. 4 presents the data from the two studies, with launch latencies from the onset of the sentence-initial noun plotted in 40 ms bins using the procedure described above in relation to the hypothetical data depicted in Fig. 2.^2^ As before, when talking of saccades launched during e.g. ‘man’ towards the man, saccades launched during ‘girl’ towards the girl are included also. In Study 1, saccades were launched towards the man during ‘man’ or ‘girl’ on 32% of all trials: of those 32% of trials, .65 were launched in response to ‘man’, and .35 in response to ‘girl’. In Study 2, saccades were launched towards the quadrant that had contained the man during ‘man’ on 17% of trials, with .70 launched in response to ‘man’, and .30 in response to ‘girl’).

It would appear in both studies that only in the first two bins (0–80 ms) were there as many saccades launched towards the man (or, in Study 2, the quadrant that had been occupied by the man) during ‘man’ as were launched during ‘girl’. In these two bins, therefore, there appeared to be no distinction between signal and noise. In subsequent bins, however, (starting 80–120 ms post onset) there appear to have been more looks towards the man during ‘man’ than during ‘girl’ (notwithstanding bin 200–240 in Study 2). The sparseness of the data renders statistical inference problematic: Although it might appear as if the data are amenable to computing a discrimination measure such as d' for each subject (and showing that in the first two bins it was not significantly different from zero, whereas in the third bin and beyond it was), the experimental design did not permit computation of a standardized d' for each participant at each bin (multiple blocks of trials would be required such that hits and false alarms could be computed for each block). Consequently, a variety of different statistical models were applied at each bin, looking for convergence amongst the models (the idea being that if different statistical models, each of which makes different underlying assumptions about the characteristics of the data, support the same conclusion about *when* (i.e. at which bin) the signal + noise and noise distributions separate, we can have greater confidence in that conclusion).

### 6.1 Study 1

Table 1 (the data from Study 1) reports two chi-square analyses at each bin (restricted to the first 10 bins only), each making different assumptions about the frequencies expected at each bin: In the first, a null hypothesis (i.e. the ‘expected’ counts contributing to the calculation of the chi-square value) is assumed in which, at each bin, looks towards the man during ‘man’ should be as frequent as looks towards the man during ‘girl’. This analysis sought to establish at which bin it first became the case that there were *more* looks towards the man during ‘man’ than during ‘girl’ (and for which it was also true that, in subsequent bins, saccades distinguished between ‘man’ and ‘girl’, bearing in mind, of course, the design of the study which ensured that each auditory token served as both signal and noise, and that each object served as both target and competitor).^3^ The second analysis assumed a null hypothesis in which, at each bin, the proportion of looks towards the man during ‘man’ or during ‘girl’ should mirror the overall proportions (i.e. .65 in response to ‘man’ and .35 in response to ‘girl’); in other words, we know that signal *will* be distinguished from noise, so the null hypothesis here is the exact opposite of that for the preceding analysis (which assumed we will *not* distinguish signal from noise) – which is that we distinguish signal from noise throughout the word. This second analysis sought to establish during which bins the observed proportion mismatched the expected proportion. If our informal inspection of the data generalizes, there should be such a mismatch in the first two bins, but not in subsequent bins (this analysis on its own would overestimate the point at which signal actually is distinguished from noise, given that the proportions that enter into the null hypothesis include data from all bins, including those where there was no such distinction).

Table 1 also includes two further statistics at each bin: partial association Likelihood Ratio Chi-Squares (LRCS) based on hierarchical log-linear models (see Scheepers, 2003), and repeated measure *t*-tests. Both these statistics are calculated on the by-participant data, and therefore determine whether the overall differences (seen in Fig. 4) generalize across participants. In the case of log-linear models, consistency across participants in the magnitude of the effect can be determined by exploring interactions between participants and the observed frequencies of signal + noise and noise responses (if the interaction term is nonsignificant, the observed frequencies are consistent across participants – see Altmann & Kamide, 2007, fn 1). The *t*-tests should be interpreted with caution given that the very small number of observations per participant violates the assumption that the data fit a normal distribution (see below for discussion of the inevitable sparseness of these data); the *t*-test is included here to allow comparison with the range of other statistical models that were applied to the data.

The first chi-square model in the table confirms our informal inspection of the data – the first bin at which looks to the man on hearing ‘man’ diverge significantly from looks to the man on hearing ‘girl’ is the bin at 80–120 ms from word onset. This divergence is statistically significant subsequently at 120–160 ms, is marginally significant at 160–200 ms, and is then significant again in the four subsequent bins. The second chi-square model gives a similar picture: In the first two bins, there is a significant difference between the observed proportion of looks to the man on hearing ‘man’ or ‘girl’ and the expected proportion (.65 and .35 respectively). In the following three bins, starting at 80–120 ms, the observed proportions are not significantly different from the expected proportions. That is, given the overall frequencies of looks to the man in response to ‘man’ or ‘girl’, the first two bins deviate from this frequency distribution but the remaining bins do not (at 200–240 ms, the deviation is significantly different, but in the expected direction – that is, significantly more looks in response to ‘man’ than in response to ‘girl’). The log-linear models reveal a similar picture, with bin 80–120 showing a significant difference in looks due to ‘man’ and looks due to ‘girl’, a pattern that is repeated in subsequent bins (albeit marginally in bin 160–200). The magnitude of each effect at each bin was consistent across participants (all *p* > .3). The *t*-tests reveal a picture that is largely similar (although the pairwise comparisons at bins 80–120 and 120–160 just fail to reach the .05 criterion).

The analyses summarized in Fig. 4 and Table 1 represent just 487 trials amongst 64 participants – each participant thus contributed an average 7.6 trials to the analysis. Divided amongst the different 40 ms bins, the data are undoubtedly sparse; it is for this reason that several different statistical models were applied to the data (linear mixed effects models are a special case of the Generalized Linear Model on which hierarchical log-linear models are based, and they would be unlikely to yield different statistical patterns unless additional predictors were to be included). From a practical perspective, replication is perhaps as important as the derivation of inferential statistics (if not more so, unless one is particularly concerned with effect size or its equivalent), and it is with this in mind that the results of Study 2 are turned to next.

### 6.2 Study 2

Fig. 4 (lower panel) shows that the pattern observed in Study 1 replicated in Study 2, even though in this second study, the scene had been removed prior to the onset of the spoken stimulus. The data from Study 2 are even more sparse than those from Study 1, as there were fewer saccades overall (this is generally the case in studies where the screen is blank at the time of the unfolding language). The 30 participants each contributed just 3.4 trials to the analysis (given the 102 trials on which saccades to the target region were launched during ‘man’ or ‘girl’); statistical generalizability is therefore unlikely. Indeed, on a per-bin basis, the assumptions underlying chi-square were violated (the majority of expected values are less than 5), and consequently chi-square analyses were not calculated for Study 2. For the log-linear models, bin 80–120 showed a significant difference between looks due to ‘man’ and ‘girl’ (LRCS = 4.0, *p* < .05), but the difference in the subsequent three bins failed to reach significance (all *p* > .1 – the differences in bin 240–280 and the subsequent two bins were significant; all *p* < .05). For the *t*-tests, bin 80–120 approached significance (*t*_(29)_ = 1.98, *p* = .06), but no other bin was significant except for 280–320 and 320–360 (*p* = .03 and *p* = .02 respectively).

### 6.3 Studies 1 and 2 combined

Given the evident similarity between the two data sets as shown in Fig. 4, and the problem of sparseness they present, a third set of analyses was conducted in which the data from the two studies was combined (if the data in Study 2 were effectively noise, this should render the statistical fits to the combined data less robust than the fits to the single Study 1 dataset, but if the data reflect the same underlying distributions as were observed in Study 1, the statistical fits should be at least as good as those to Study 1 alone). The statistical analyses corresponding to this combined data set are presented in Table 2. The two chi-square models, the log-linear models, and the *t*-tests, converge on the same interpretation: at the first two bins, there is no difference in looks to the man as a function of hearing either ‘man’ or ‘girl’. At the third bin (80–120 ms) and subsequently, there are significantly more looks towards the man in response to ‘man’ than in response to ‘girl’. In respect of the log-linear models, the magnitude of the difference between looks due to ‘man’ and looks due to ‘girl’ was consistent across participants at each bin (all *p* > .9). It is possible to conclude from the combined data, therefore, that signal-driven eye movements distinguish from noise-driven eye movements between 80 and 120 ms after word onset. We can also conclude, albeit very tentatively, that this pattern holds across both data sets separately (to the extent that adding the sparse data from Study 2 to the Study 1 data rendered the statistical model fits more robust).

## 7 Discussion

Despite the sparseness of the data, the fact that multiple statistical models converge on the same pattern suggests the validity of the conclusion: language-mediation of oculomotor control can occur within 80–120 ms; examination of the distribution of launch times towards the man (or the girl) as the words ‘man’ or ‘girl’ unfold shows that there were more signal-driven saccades launched in this and subsequent bins than there were noise-driven saccades. This would appear to have been the case not just when the scene was concurrent with the unfolding word (Study 1) but also when the scene had been removed prior to the spoken stimulus (Study 2, although interpretation of these data is hampered by their sparseness). Unlike prior estimates of the time-course with which language can influence oculomotor control, these studies were typical ‘visual world’ studies in which the eyes were free to fixate on any part of the scene prior to the onset of the target word. The speed with which language-mediation was observed in these studies is all the more remarkable given the nature of the *look-and-listen* task that was employed in both studies: participants were not under instructions to move their eyes as quickly as they could – indeed, they were under no explicit instruction at all. Whether more explicit task demands would change the patterns observed in these studies is an empirical question; most likely the timing with which signal is distinguished from noise would not change, but the number of saccades would.

If it takes around 200 ms to plan and launch a saccade (given the earlier estimates of saccadic latency), how could one possibly observe language-mediated oculomotor control in just 100 ms? One possibility has to do with the fact that in these studies, the critical target onset (the /m/ in ‘man’ or /g/ in ‘girl’) was preceded by a determiner (‘the’) which may convey, on its vowel, coarticulatory information about either manner or place of articulation of the following consonant. The same is true of the Allopenna et al. (1998) study (coarticulation on ‘the’ in ‘the beaker/beetle’ would have been distinct from ‘the’ in ‘the carriage’, for example), and yet their data showed a 200 ms delay from the onset of the *noun* before eye movements towards the beaker or beetle diverged from eye movements towards the baby carriage. Thus, to the extent that the studies reported here and in Allopenna et al. (1988) had comparable coarticulation, it is unlikely that the fast effects observed here are due to that coarticulation. A subsequent study by Dahan, Magnuson, Tanenhaus, and Hogan (2001) demonstrated that the eye movement record *is* sensitive to coarticulatory information: They compared the eye movement record in response to words such as ‘net’ (where the first consonant and vowel were taken from a different articulation of this same word) and a version of ‘net’ where the first consonant and vowel were taken from an articulation of the word ‘neck’. They found that this difference manifested in the eye movement record an average of 220 ms after the offset of the initial consonant-vowel sequence (i.e. the onset of the final consonant that disambiguated the unfolding sequence as ‘net’). In Studies 1 and 2, the corresponding point would be the offset of the determiner (i.e. the onset of ‘man’ or ‘girl’), in which case the effects of coarticulatory information could not be expected to manifest, given the Dahan et al. (2001) finding, until around 220 ms after the onset of the target word – in other words, coarticulatory effects would have little impact on the eye movement record within the 100 ms timeframe identified in Studies 1 and 2. Moreover, Dahan et al. (2002) manipulated coarticulatory information *within* the target word – it is an empirical question whether coarticulatory information on a determiner can cause eye movements towards just those objects in the scene whose names might be compatible with that coarticulatory information (but see below for further discussion of what effects coarticulation could have within the timeframe suggested by Studies 1 and 2).

Given that coarticulation is an unlikely explanation for the speed with which signal was distinguished from noise in Studies 1 and 2, a further possibility is that the data are due to *express saccades* (e.g. Cavegn & d'Ydewalle, 1996; Fischer & Boch, 1983; Fischer & Ramsperger, 1984) – visually-guided saccades with short latencies of around 100 ms However, to our knowledge, such saccades have only been observed when the signal to move the eyes is the onset of a visual target. A more likely possibility is that the effects observed here are not due to the rapid planning and subsequent launching of eye movements, but are instead due to the *cancellation* of already-planned saccades towards some object *other* than the one that the unfolding language will refer to. Estimates of the time required to cancel a saccade vary between around 100 ms (Kornylo, Dill, Saenz, & Krauzlis, 2003) and 160 ms (Curtis, Cole, Rao, & D'Esposito, 2005), although these are averaged estimates, and do not reveal the likely distribution of cancellation times; nonetheless, the time-course of cancellation is compatible with the data from Studies 1 and 2. But even disregarding the time it takes to either plan or cancel a saccade, 100 ms is barely time to know what the unfolding word will become (depending on the initial phonemes, 100 ms may or may not include a part of the vowel following the first phoneme, although as indicated earlier, co-articulatory information conveyed by the preceding determiner – ‘the man’ – may also provide information about the identity of the first phoneme). Given the rapidity of the observed effects, and the minimal phonological information conveyed by those first 100 ms, the following is one possible account of the data.

As in Altmann and Kamide's (2007) account of why the eyes move in the visual world paradigm, it is assumed that free inspection of the visual scene activates conceptual representations of the objects contained within the scene. A part of this conceptual knowledge is the phonological specification of the object's possible names (see Meyer, Belke, Telling, & Humphreys, 2007, for an example of how phonological information is activated even during a visual search task for which such information is task-irrelevant). It is further assumed that eye movements towards an object that has already been identified are preceded by shifts in covert attention towards that object (e.g. Henderson, 1992; Henderson, Pollatsek, & Rayner, 1989), and that these pre-saccadic shifts (which under the premotor account of attention constitute the saccadic plan) re-activate conceptual knowledge about those objects *including* that phonological knowledge about the objects' names (it is conceivable that aspects of such knowledge are available from parafoveal preview alone – in the studies above, the man and the girl were the only animate objects, and were thus particularly salient and highly likely to have been fixated in the earliest moments of the scene's presentation; the current data set do not allow contingent analyses on participants *not* having previously fixated the target objects). Thus, when planning a saccade towards e.g. the girl, phonological knowledge about the targeted object is known in advance of launching the saccade; in effect, we know the name of the thing we're about to launch an eye movement to. If, simultaneously, we hear the /m/ in ‘man’ (preceded, perhaps, by coarticulatory information about the upcoming /m/) we cancel the saccade because of the phonological mismatch between the unfolding word (‘man’) and the name of the currently targeted object (‘girl’). Canceling eye movements towards the girl when we hear the earliest moments of ‘man’ (or towards the man when we hear the earliest moments of ‘girl’), or alternatively, canceling eye movements when we can anticipate the upcoming /m/ on the basis of coarticulation on the preceding vowel, would lead to the observed pattern of data: Consider, for example, two hypothetical participants, both of whom have been fixating some location within the scene for exactly 150 ms. Participant A is planning a saccade to the man (and will execute that saccade in, say, another 100 ms); Participant B is planning a saccade to the girl (which again, would be executed in another 100 ms) 150 ms into their respective fixations they hear ‘man’ – Participant A continues with the planned saccade, but Participant B cancels their own planned saccade. Thus, 100 ms after the onset of ‘man’, Participant A launches towards the man, but Participant B does *not* launch towards the girl – hence one more saccade at 100 ms to the man than to the girl on hearing ‘man’ (and perhaps for another pair of participants, to the girl than to the man on hearing “girl”). This hypothetical example, mirroring the behavioral data, demonstrates language-mediated discrimination between intended and unintended targets at 100 ms; not because more saccades are launched towards the intended target, but because fewer saccades are launched towards the unintended target.

This is, of course, conjecture: a number of testable predictions follow, however – for example, increasing the number of phonological competitors that share that same first phoneme should lessen the effects (in the example given for Study 1, eye movements planned towards the motorbike would not be cancelled, for example. Across the different items in that study, there were insufficient trials with such competitors to test the prediction).

There is an alternative account of why saccades might be cancelled: Prior inspection of the scene leads to the activation of conceptual representations which prime their subsequent reactivation by the partially unfolded phonetic input; having seen the girl means that the /g/ in ‘girl’ is interpreted as indicating with greater likelihood (than if heard out of this visual context) the word “girl”. In other words, the previously seen girl primes the conceptual representations that are compatible with that initial /g.../ sequence, and the representational ‘boost’ that accrues due to the overlap between the conceptual representations activated by the unfolding speech and the conceptual representations previously activated by the girl constitutes a change in attentional state that accompanies saccadic planning (see the earlier discussion of interpretation, attention, and planning, as well as Altmann & Kamide, 2007, for discussion of this representational boost). This suggests that the mismatch that causes cancellation of the saccade towards the man could be due not simply to *phonological* mismatch, but to *conceptual* mismatch also; the /g/ in ‘girl’ activates the conceptual correlates of the word, given that these have been primed by the girl in the scene, and the mismatch between this conceptual representation and the conceptual representation that is concomitant with the planned eye movement towards the man causes that eye movement to be cancelled. The current dataset cannot determine whether phonological or conceptual mismatch (or both) drives the cancellation process, although the greater rapidity in the visual world paradigm with which phonological effects can be observed relative to semantic effects (e.g. Huettig & McQueen, 2007) suggests that, at the very least, the earliest cancellations are likely to be driven by phonological mismatch.

This same account applies also to the blank screen data (Study 2, although the sparseness of the data rendered that study less conclusive): Hearing the earliest moments of /g/ activated the conceptual representation corresponding to the word “girl” (because of the priming afforded by the previously seen scene, as described above), and the activation of this conceptual representation causes the initiation of a plan to move the eyes back towards the location previously occupied by the girl. The planning of this subsequent eye movement necessarily competes with the planned eye movement that is about to be launched elsewhere, resulting in the inhibition and more likely cancellation of the originally planned eye movement. An account based on phonological match/mismatch is also possible if we assume that phonological information, and not just conceptual information, is associated with each location.

One substantive issue remains: How is the initially planned eye movement in fact cancelled? With respect to cancellation of saccades, the visual world paradigm resembles, at times, a *double-step* paradigm. Double-step procedures in eye-movement research involve a target appearing in one location, to which an eye movement is prepared, and shortly after, a second target appearing at a new location (depending on the task, the first target may remain onscreen or may be replaced by the second). If the interval between the two steps is short (around 100 ms), participants will often inhibit the planned saccade to the first target and respond with a single saccade toward the second target – accrual of information in one channel blocks, or (in more recent formulations) competes with (and can therefore inhibit), processing in another; e.g. Becker and Jurgens (1979, see Ray, Schall, & Murthy, 2004, for an account of double-step saccade sequences compatible with the guided activation theory of cognitive control cited earlier, and Findlay & Walker, 1999, for an account of saccade generation based on competitive inhibition). In the visual world paradigm, the equivalent situation occurs when an eye movement is planned towards one location (the first step), but the unfolding language suggests another location (the second step); in the cases reported here from Studies 1 and 2, the first step corresponds to the endogenously cued location towards which the eyes are initially programmed, regardless of the language, and the second corresponds to the location exogenously cued by the earliest moments of the language (this is not the only way in which the double-step concept applies to the visual world paradigm – later in the sentence, both steps could be linguistically mediated, or other exogenous or indeed endogenous processes could cause double-step-like behavior). Within this double-step interpretation of the data, cancellation of saccades is a natural behavioral phenomenon (another phenomenon associated with competition between alternative locations to which a saccade might be directed concerns saccadic curvature (e.g. Walker, McSorley, & Haggard, 2006), although saccadic trajectories were not assessed in the current study).

This raises, however, a secondary, but equally important issue: in the double-step paradigm, inhibition of the initially planned saccade is due to an alternatively cued target that triggers an alternative plan, but in the account of the present data given above, this inhibition is due to *mismatch* against information associated with the initially planned saccade. On the face of it, these are two very different phenomena. However, an alternative (competition-based) perspective on the double-step phenomenon is that the accrual of information during the planning of the first saccade, but which pertains to the planning of the second step (cf. parallel programming of saccades; Walker & McSorley, 2006), inhibits the first saccade precisely because it mismatches the information that constitutes the plan for the first saccade. To return to the data above: The mismatch that arises between /m/ and the phonological information associated with the targeted location to which the initial saccade is planned is accompanied by a *match* between the /m/ and the phonological information associated with other locations in the scene (although recall that the match/mismatch could also arise at the conceptual level). The visual world paradigm relies, generally, on the fact that the objects in the scene are already known by the time the language comes along – activating conceptual representations in response to a word such as ‘girl’ causes the eyes to move towards the girl in the scene precisely because the location of that girl is already known (again, see Altmann & Kamide for a mechanistic account of this process). Thus, *mismatch* in the visual world paradigm (assuming that the sentence does felicitously apply to the depicted scene) is necessarily accompanied also by *match*. Hence the competitive planning processes which, in the more typical double-step paradigm, result in cancellation or inhibition of the initially planned saccade (and which might result also in influences on saccadic curvature).

The account of how it is that signal can be distinguished from noise so early in the language-mediated eye movement record also explains why the earliest data are necessarily sparse – this early mediation, through cancellation of an initially planned saccade, will only occur on those trials in which, fortuitously, the target word unfolds at just the right moment in time relative to that planned saccade (most likely that unfolding must precede the onset of the originally planned saccade – if the unfolding occurs while the eye is executing the saccade (or too late for that saccade to be cancelled), the early influence of the language may manifest differently, as a brief fixation on the target location followed by a corrective saccade whose programming preceded that brief fixation). Thus, cancelled saccades will likely be considerably rarer than executed saccades (although within the visual world paradigm this remains an empirical issue, and will depend on many different factors including, for example, whether or not the language is highly predictive of what will be referred to next; saccadic launch times, for example, are known to be dependent on the number of alternative locations to which a saccade may need to be launched and the informativeness of the signal (Carpenter & Williams, 1995; Reddi et al., 2003) and thus is it likely that the effects observed here would change as a function of e.g. scene complexity and informativeness of the language – cf. the earlier prediction about the number of phonological competitors in the scene).

If the data from Studies 1 and 2 are indeed due to cancellation of saccades (cf. the double-step paradigm), rather than to saccadic planning *de novo*, they have implications for the interpretation of other visual world data: First, the hypothesis that links eye movements to changes in cognitive state due to the unfolding language (Altmann & Kamide, 2007; Tanenhaus, Magnuson, Dahan, & Chambers, 2000) has to be modified to include cancellation of pre-programmed eye movements, such that rapid effects of mismatch and match (as in the studies by Allopenna et al., 1998, and others) could manifest in cancelled eye movements. Second, the widely-held assumption that eye movement patterns within the first 200 ms following some part of the linguistic signal could not be due to that signal need also to be modified: While it may be true (on the basis of psychophysical studies of oculomotor control) that *on average* eye movements cannot be planned *de novo* and executed within much less than 200 ms, they can be *cancelled* well within that 200 ms timeframe, and the fixation/saccadic record will be influenced by those cancelled saccades. The data reported here suggest that, if it *is* necessary to make any assumptions at all about when the language can first influence the eye movement record, this timeframe must be halved to 100 ms. Finally, the necessary theoretical confluence of saccadic planning and interpretation of the unfolding language, together with the observed speed with which signal-driven saccades distinguish from noise-driven saccades, suggests that the mediation of the one process (saccadic planning) by the other (language interpretation) is *automatic*, in the sense that it is not under conscious volitional control (although this does not entail that it is task-independent – that remains an empirical issue); in support of this claim, most theories of saccadic control maintain that the modification of existing saccadic plans is not under volitional control (e.g. Findlay & Walker, 1999).

Further research is clearly needed: As indicated above, the claim that these data are due to cancellation of saccades is conjecture, as is the (associated) parallel that has been drawn between certain kinds of saccadic behavior in the visual world paradigm and saccadic behaviors observed in the double-step paradigms that have been used to study saccadic control. Moreover, this was an opportunistic reanalysis of earlier studies that had not been designed to address specifically the issues described here. A considerably larger dataset is required for a more in-depth analysis of what in fact is driving the effects reported here. The fact that we observe language-mediated oculomotor control within around 100 ms in a task which does not explicitly encourage rapid behavioral responses means that further data are required to explore also how this time-course might change as a function of task, and how it might change as a function of the complexity of the scene and the phonological and conceptual characteristics of the objects depicted within the scene. Many other parameters may be relevant also, such as the time to preview the scene before the onset of the critical signal, the clarity of that signal, where the eyes are fixating as that signal unfolds (or whether they are in motion), and so on. For now, it remains the case that across two separate studies with very different visual properties (the scene being concurrent or absent), and across a range of different statistical models (making different assumptions about the underlying data), there is substantial convergence on the time-course with which language can mediate oculomotor control – within 100 ms.

## Figures and Tables

**Table 1:** Study 1: Four different statistical models of the data depicted in Fig. 4 (top panel). * *p* < .05, † *p* < .1.

Model statistic	Chi-square: signal equals noise at each bin		Chi-square: signal/noise at each bin matches overall signal/noise ratio		Hierarchical log-linear models (testing for a difference between signal and noise at each bin)		Repeated measure *t*-tests (testing for a difference between signal and noise at each bin)	
	*χ*^2^	*p* (*df* = 1)	*χ*^2^	*p* (*df* = 1)	LRCS	*p* (*df* = 1)	*t*	*p* (*df* = 63)
Bin								
0–40	0.0	0.90	4.9	0.03*	0.0	0.89	0.1	0.91
40–80	0.3	0.62	4.0	0.05*	0.3	0.62	0.5	0.64
80–120	3.9	0.05*	0.0	1.00	4.0	0.05*	1.9	0.06^†^
120–160	5.7	0.02*	0.0	1.00	5.8	0.02*	2.0	0.05^†^
160–200	3.3	0.07^†^	0.3	0.58	3.3	0.07^†^	2.2	0.03*
200–240	17.2	0.00*	4.5	0.03*	18.3	0.00*	3.9	0.00*
240–280	6.1	0.01*	0.6	0.42	6.3	0.01*	2.8	0.01*
280–320	6.1	0.01*	0.4	0.51	6.3	0.01*	3.1	0.00*
320–360	6.8	0.01*	1.3	0.25	7.1	0.01*	2.6	0.01*
360–400	2.6	0.11	0.1	0.76	2.6	0.10	1.5	0.15

**Table 2:** Studies 1 & 2 combined: Four different statistical models of the data depicted in Fig. 4 (both panels). * *p* < .05, † *p* < .1.

Model statistic	Chi-square: signal equals noise at each bin		Chi-square: signal/noise at each bin matches overall signal/noise ratio		Hierarchical log-linear models (testing for a difference between signal and noise at each bin)		Repeated measure *t*-tests (testing for a difference between signal and noise at each bin)	
	*χ*^2^	*p* (*df* = 1)	*χ*^2^	*p* (*df* = 1)	LRCS	*p* (*df* = 1)	*t*	*p* (*df* = 93)
Bin								
0–40	0.0	0.90	5.9	0.01*	0.0	0.90	0.1	0.91
40–80	0.1	0.73	6.2	0.01*	0.1	0.73	0.3	0.73
80–120	6.5	0.01*	0.1	0.75	6.6	0.01*	2.5	0.01*
120–160	7.5	0.01*	0.0	0.95	7.6	0.01*	2.3	0.03*
160–200	4.5	0.03*	0.4	0.55	4.6	0.03*	2.4	0.02*
200–240	14.5	0.00*	1.9	0.17	15.1	0.00*	3.6	0.00*
240–280	10.1	0.00*	1.1	0.30	10.5	0.00*	3.2	0.00*
280–320	10.1	0.00*	1.1	0.30	10.5	0.00*	3.8	0.00*
320–360	12.6	0.00*	3.1	0.08^†^	13.5	0.00*	3.6	0.00*
360–400	3.2	0.07^†^	0.1	0.82	3.3	0.07^†^	1.7	0.10^†^

## Footnotes

1 Eye movements towards the man (or its equivalent across trials) during ‘girl’ (or its equivalent) could be due to the semantic overlap between the conceptual structure activated by ‘girl’ and that activated by seeing the man (e.g. Huettig & Altmann, 2005; Yee & Sedivy, 2001, 2006) – this possibility is ignored for the present. Further research would be required to establish whether the time-course data reported below are influenced by this overlap.

2 The data were initially analysed using software originally developed in collaboration with research groups at Glasgow and Saarbrucken. To check for accuracy, the raw EDF files from Study 1, which were output by the EyeLink system used for both studies, were imported into the SR-Research DataViewer package (used with subsequent EyeLink II systems) and reanalysed using DataViewer. Slight differences, which did not change the observed patterns, emerged through the re-drawing of the regions of interest that was necessary for re-analysis by DataViewer. Some automated analysis schemes routinely incorporate brief fixations (< 80 ms or < 100 ms) into the following fixation; thus, the onset of a saccade that landed on the target region which had been preceded by such a short fixation would be calculated as the onset of the *preceding* saccade (which had resulted in that short fixation). In fact, it made no difference whether or not brief fixations were treated in this way (because of the very small numbers involved). The original data, using the more accurate regions of interest, are reported here.

3 In determining the first bin at which the number of saccades in response to ‘man’ is significantly greater than the number of saccades in response to ‘girl’, the following simplifying assumptions are made: (1) *If* saccades launched in that bin are *completely independent* of saccades launched in, for example, the two successive bins, and if the *p*-value for the inferential statistic computed separately at each of these three bins (the first to show a significant difference, and the next two) is .049, the *p-*value associated with finding a joint effect across all three bins is .049 × .049 × .049 (= .0001). (2) If, *alternatively*, the saccades launched in those three bins are *completely dependent* on one another, and if the *p*-value computed at the first bin is .049, the *p*-value associated with finding a joint effect across all three bins is also .049 (there is a further assumption here that the dependence works forwards in time – that is, saccades at bin *n* are not dependent on bin *n +* 1, rather, those at bin *n +* 1 are dependent on those at bin *n*). (3) Without in fact knowing the *degree* of dependence between the three bins (or however many we choose to analyze in this way), we must assume that the correct *p*-value (correcting for some degree of dependence) lies in the range .05 > *p* > .05^*n*^, where *n* is the number of bins we analyze in this way. This is similar, in some respects, to the application of cluster-based Gaussian logic (e.g. Worsley, Evans, Marrett, & Neelin, 1992) employed in neuroimaging (as an alternative to applying Bonferonni correction for the multiple comparisons made across neighboring voxels, a correction that is likely inappropriate given the likely dependence between such voxels), although a Gaussian fit across neighbouring bins is not applied here. This same logic can be used, in principle, to determine at what point two curves plotting e.g. probability of fixation diverge (and is particularly useful when the difference between the curves increases monotonically, as is more likely in a task that involves reaching towards real or virtually displayed objects; where the difference is non-monotonic, as in the Gaussian logic, an arbitrary threshold must be set for the number of adjacent comparisons that will be computed in order to estimate the lower bound on the *p*-value).
